# A Bibliometric Analysis of Robotic Surgery From 2001 to 2021

**DOI:** 10.1007/s00268-022-06492-2

**Published:** 2022-03-08

**Authors:** A. Musbahi, C. B. Rao, A. Immanuel

**Affiliations:** 1 Northern Oesophago-Gastric Unit, Royal Victoria Infirmary, Newcastle, UK; 2 UCL Medical School, University College London, 74 Huntley St, Bloomsbury, London, WC1E 6DE UK

## Abstract

**Introduction:**

Bibliometric analyses are a method of evaluating the quality of research output in a certain domain. Robotic surgery has made vast leaps during the past 20 years and this paper aimed to assess some of the main areas of research using this method.

**Methods:**

A search was undertaken for documents published between 2001 and 2021 from the World of Science database, using the keywords ‘robotic surgery’, ‘robotic assisted surgery’ and ‘robotic-assisted surgery. Results were compared using numerous bibliometric methodologies, and stratified by source-specific metrics, author-specific metrics and country-specific metrics.

**Results:**

The search yielded 3839 documents, from 879 different sources. Only 2% of sources were found to be within Bradford’s Zone 1 of research and the most relevant sources were from the field of urology. The Journal of Urology and Surgical Endoscopy and other Techniques ranked highly among metrics such as H, G, M index and total citations. The top-rated authors had a *H* index of 15 in the field of robotic surgery and the total citations reached a peak at 1342. The USA, Japan and Italy were the most productive nations and increased collaborative research is leading to a greater number of multiple-centre publications.

**Conclusion:**

Research into robotic surgery is still in its infancy with further reviews of the literature and greater output through large randomised controlled trials in multiple centres through collaborative research needed.

## Introduction

Robotic surgery has advanced significantly in the last 20 years. Whilst beginning as stereotaxic systems in the late 1980s, for example the PUMA 200 [1], surgical robots have adapted to not only enable surgery with fewer cuts, but with better precision, accuracy, degrees of freedom and even magnification. A steady movement towards a fifth generation of autonomous robot is being made [2].

Specialties such as urology and gynaecology have long been trailblazers in robotic application and research with some expansion into general surgery and cardiothoracic surgery. The future frontiers of robotic surgery and its full capability are yet to be realised.

Research in robotic surgery was led with the publication of seminal works, such as Nix et al.’s randomised clinical trial on radical cystectomy [3]; however, the research field is young with higher levels of evidence required to prove equivalence or benefit over standard laparoscopic or open techniques.

Bibliometric analyses are defined as efforts to evaluate the quality of research through the measurement of various parameters of scholarly output. This enables a reader to gauge not only the volume of an author’s output or the rate, but an objective demonstration of the number of citations and relationships between authors and articles, not limited to peer-reviewed manuscripts. This then allows analysis of the impact and popularity of publications, authors, institutions and collaborative links.

This analysis can be used with numerous applications, including grant allocations, and by policymakers to set standards for research and direct suitable funding. This study aims to use bibliometric techniques to identify the research trends and patterns of robotic research output from the last 20 years (2001–2021).

## Materials and methods

A comprehensive search of the literature was completed. This was taken from the ‘Web of Science Collection’, a subset of the ‘Web of Science’ database, which includes the Science Citation Index Expanded (SCIE), the Social Sciences Citation Index (SSCI) and the Arts and Humanities Citation Index (A&HCI). This database was chosen due to its standing in the academic world as one of the premier citation search platforms [4] and has been proved to be more accurate than rivals such as Scopus in fields such as its journal classification system [5, 6].

A search using the keywords, ‘robotic surgery’, ‘robotic assisted surgery’ and ‘robotic-assisted surgery’ was performed between January 2001 and January 2021.

After completing this search source-specific metrics, author-specific metrics and country-specific metrics were found. A keyword analysis of all the sources was also performed.

### Source-specific metrics

The relevance of a source was measured using the total number of documents drawn from a source and were then clustered, using Bradford’s Law into zones. Zones as shown by Bradford’s Law as are indicative of their utility in a certain field [7, 8]. In this case, journals or sources in Zone 1 would be those with the highest productivity within robotic surgery and would represent “the core” of the literature. Total citations (TC) and the number of documents drawn per year per source were also included.

### Author-specific metrics

The impact and relevance of authors were considered by drawing their number of documents with an absolute and a fractionalised value, used to understand their contributions in the context of both individual and collaborative research. Fractionalised counting allocates the credit of publication to co-authors in a fractional way, thus by comparing the total and fractional number of articles, one can analyse both participation and contribution to the field of robotic surgery, respectively [9, 10]. Total citations per author were also considered, along with the *h*, *g* and *m* index values. The *h*-index is a value that combines both publication and citation count to form a sole value. If an author publishes five articles, each with five citations, his/her *h* index would be 5. This would only increase if he/she published a sixth article and their total articles managed a minimum of six citations each. This begins to quantify both the quality and quantity of an author’s productivity [11]. The *g* index of an author is another metric of output, which is defined as an author’s top g articles that have been cited an average of *g* times or at least *g*^2^ times. The M index is an author’s *H* index/the years since their first publication. This tries to compare the output levels of author over time and takes into account early researchers.

### Country-specific metrics

The number of documents per country, the number of single country publications (SCP) and their involvement in multiple country publications (MCP) was considered. An MCP ratio was calculated, which indicates the level of international collaboration in an evidence base. The number of countries involved is calculated as a ratio of the total number of publications with the first author being from that country.

### Statistics

Data were collected and collated on Excel (*Microsoft, United States*). Statistical analysis was completed using IBM SPSS Statistics *(IBM, United States)*.

## Results

### Demographics of the literature

A total of 3839 documents were found and analysed, from 879 different sources. A summary of this data is shown in Table 1. 13,378 different authors were involved in this research, 99.1% (13,258) of which were part of multi-authored documents. On average, there were 3.48 and 5.05 authors and co-authors, respectively, per document, with a mean of 0.287 documents per author. The mean Collaboration Index was 3.58. Furthermore, 68.1% (*n* = 2613) of all studies were articles, with the rest of the documents spread between book reviews, editorial materials, letters, proceedings papers, abstracts, news items, reprints and reviews.

**Table 1:** Collection of overarching information regarding the collection

Main information about the collection	Description	Results
Main information about data	Timespan	2001–2021
	Sources (Journals, Books, etc.)	879
	Documents	3839
	Average years from publication	5.9
	Average citations per documents	13.75
	Average citations per year per doc	1.735
	References	1
Document Types	Article	2613
	Article; proceedings paper	144
	Book review	1
	Correction	4
	Editorial material	126
	Letter	30
	Meeting abstract	171
	News item	1
	Proceedings paper	195
	Reprint	1
	Review	552
	Review; book chapter	1
	DocumenT Contents	
	Keywords Plus (ID)	4170
	Author's keywords (DE)	5436
Authors	Authors	13,378
	Author appearances	19,401
	Authors of single-authored documents	120
	Authors of multi-authored documents	13,258
Authors collaboration	Single-authored documents	131
	Documents per author	0.287
	Authors per document	3.48
	Co-Authors per documents	5.05
	Collaboration index	3.58

**Fig. 1:**
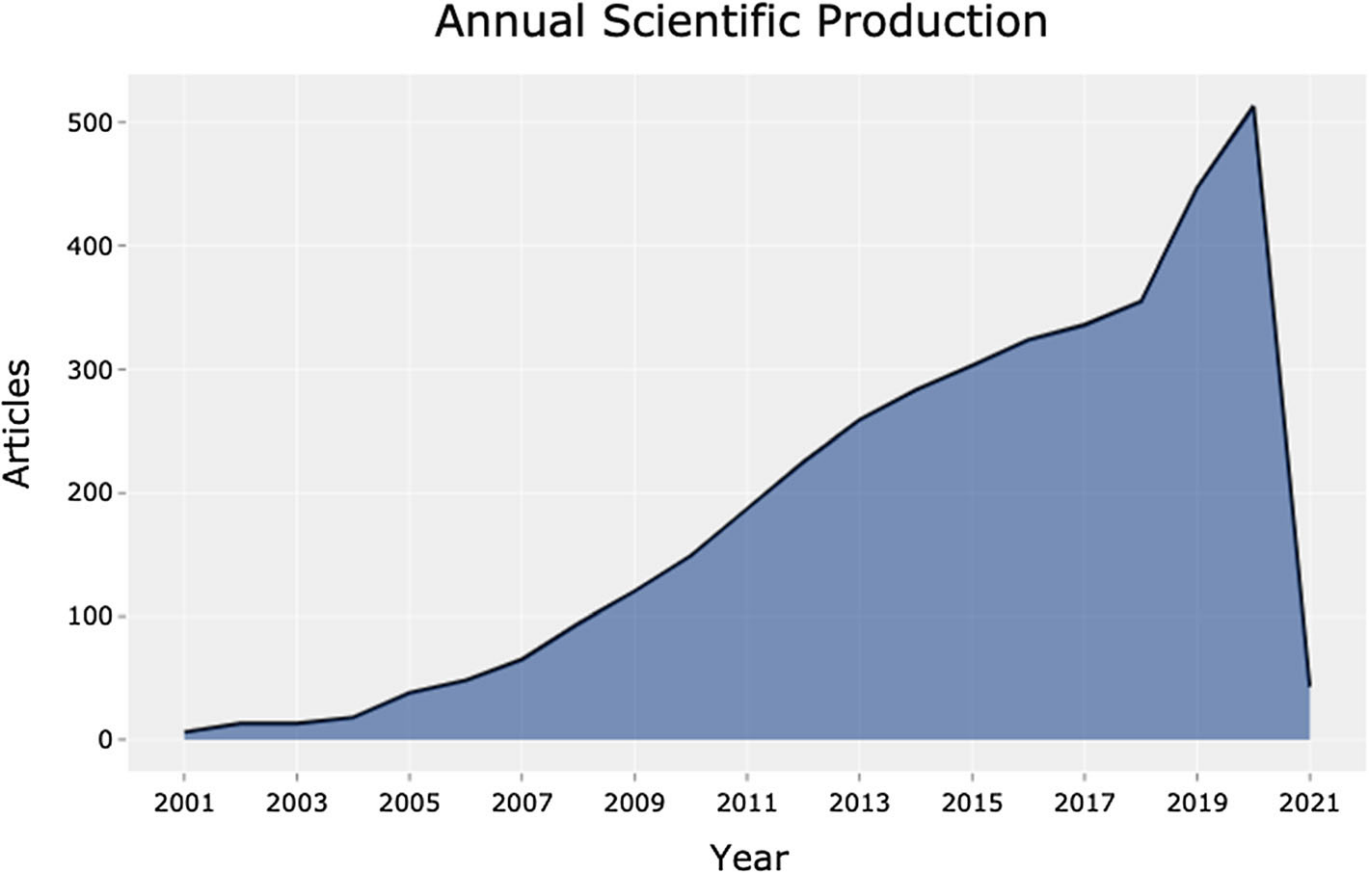
Graph showing the annual scientific production

As shown in Fig. 1 and Table 2, productivity has greatly increased over the last 20 years. Over the past 5 years, 52.6% (*n* = 2018) of the literature over the course of the past 20 years has been produced. The greatest proportional increase was between 2001–2005 and 2006–2010, with a 5.4 × increase in the number of documents created.

**Table 2:** Annual scientific productivity on robotic surgery

Year	Articles
2001–2005	88
2006–2010	476
2011–2015	1257
2016–2021	2018

**Table 3:** Table showing the resources in Zone 1

Source	Rank	Frequency	Cumulative frequency	Zone
Journal of Robotic Surgery	1	241	241	Zone 1
Surgical Endoscopy and Other Interventional Techniques	2	127	368	Zone 1
Urology	3	91	459	Zone 1
International Journal of Medical Robotics and Computer Assisted Surgery	4	87	546	Zone 1
Journal of Minimally Invasive Gynecology	5	77	623	Zone 1
Journal of Urology	6	66	689	Zone 1
Journal of Thoracic Disease	7	55	744	Zone 1
JSLS-Journal Of The Society Of Laparoendoscopic Surgeons	8	54	798	Zone 1
Gynecologic Oncology	9	53	851	Zone 1
Current Opinion In Urology	10	44	895	Zone 1
Journal Of Laparoendoscopic & Advanced Surgical Techniques	11	43	938	Zone 1
Innovations-Technology And Techniques In Cardiothoracic And Vascular Surgery	12	41	979	Zone 1
BJU International	13	40	1019	Zone 1
International Journal Of Gynecological Cancer	14	39	1058	Zone 1
World Journal Of Urology	15	36	1094	Zone 1
Annals Of Thoracic Surgery	16	35	1129	Zone 1
Journal Of Pediatric Urology	17	32	1161	Zone 1
Obesity Surgery	18	30	1191	Zone 1
European Urology	19	29	1220	Zone 1
Female Pelvic Medicine And Reconstructive Surgery	20	27	1247	Zone 1
Canadian Journal Of Urology	21	26	1273	Zone 1

### Source analysis

**Fig. 2:**
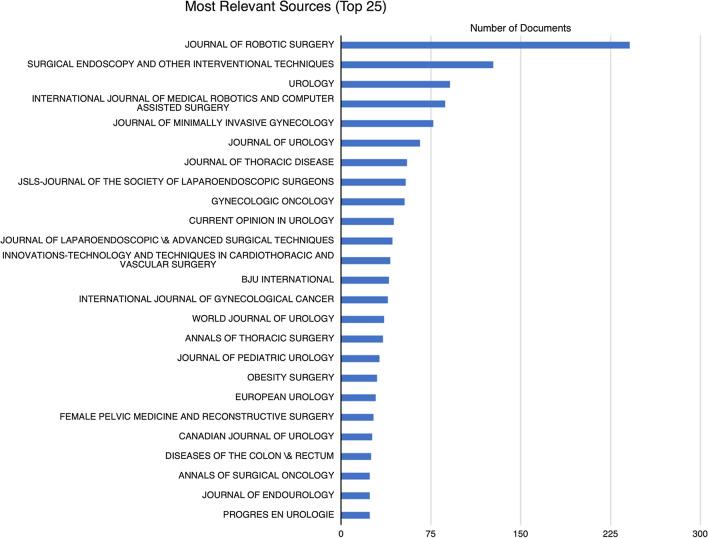
Graph showing the distribution of the Top 25 most relevant sources

Sources were ranked by relevance to the topic with the total number of documents per source. The Journal of Robotic Surgery housed the greatest number of documents (*n* = 241), followed by Surgical Endoscopy and Other Interventional Techniques (*n* = 127) and Urology (*n* = 91) as the next most relevant. The top 25 most relevant sources are charted in Fig. 2. Building on this, 40% of the 25 most relevant sources were related to the specialty of urology, with gynaecology, thoracic surgery followed by general surgery.

**Fig. 3:**
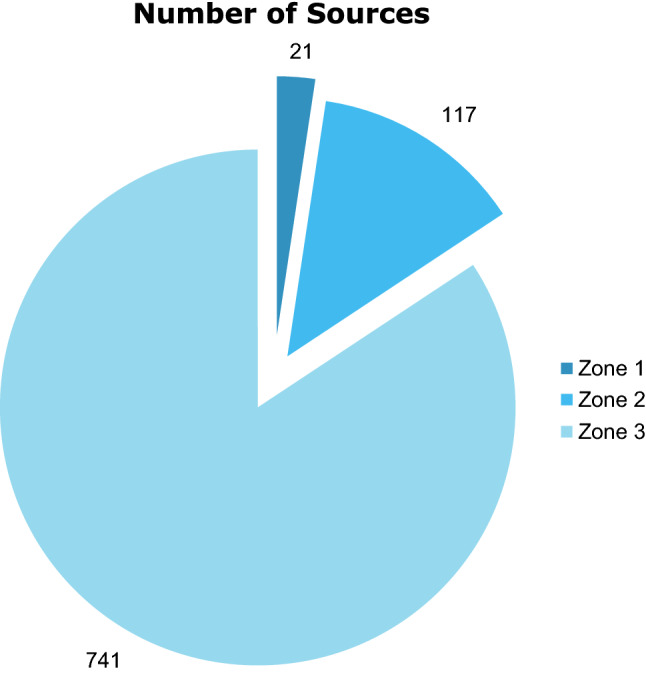
Pie Chart showing Bradford's Law

When sources were clustered using Bradford’s Law, only 2.39% (*n* = 21) sources were in Zone 1 this being the core of the literature, 1.33% (*n* = 117) were in Zone 2 and 84.3% (*n* = 741) were in Zone 3. 33.3% (*n* = 7) of the sources in Zone 1 were related to the field of urology.

**Fig. 4:**
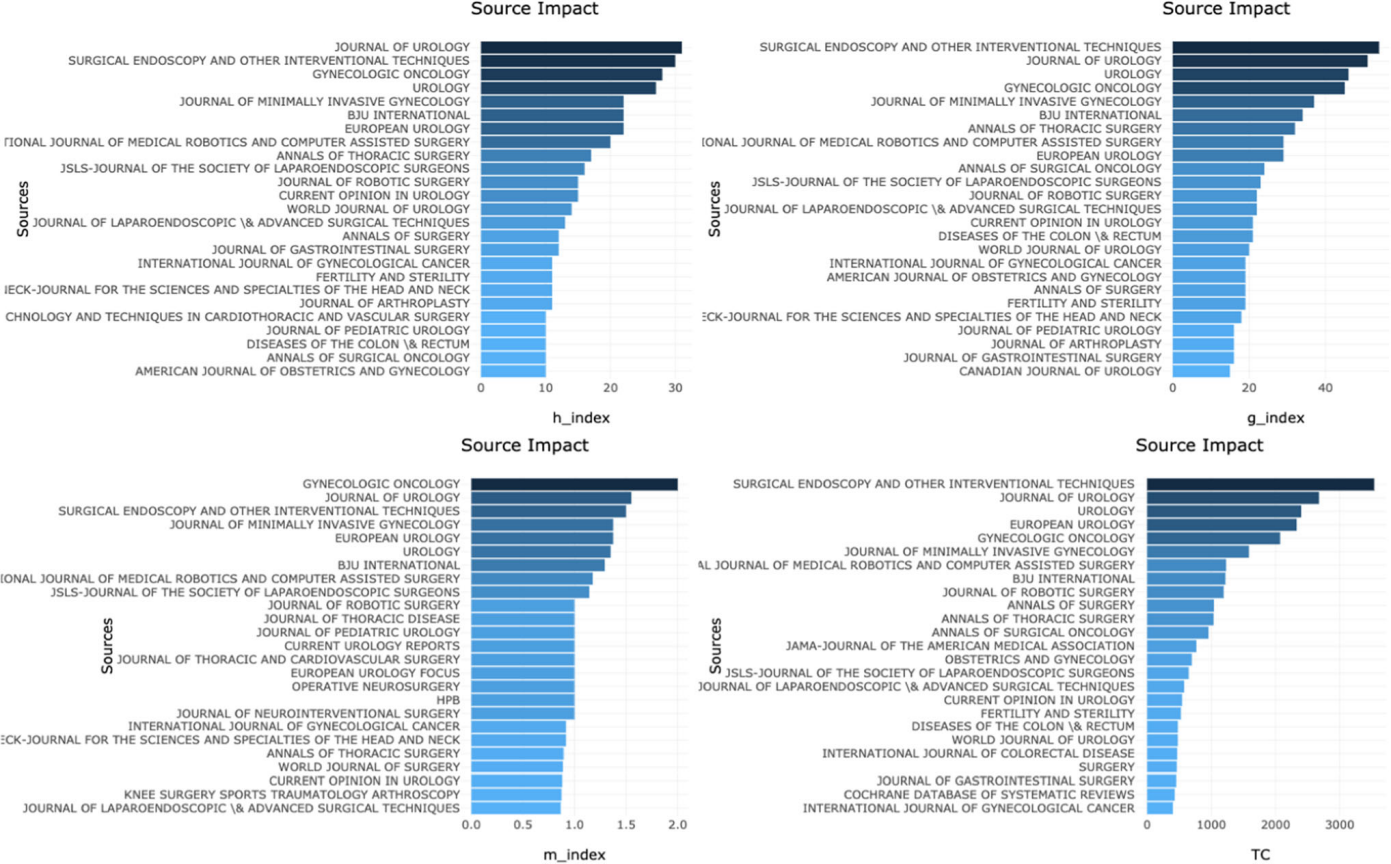
Graph showing the h index, g index, m index and total citations of the top 25 most productive sources

Subsequently, sources were stratified by their *h* index, *g* index, *m* index and total citations, as shown in Fig. 4. The Journal of Urology was well represented in all three measures, with the top-ranked *h* index, the second *g* and *m* index and total citations. This indicates that there was not only a large amount of data from this journal, but it was influential in the field. Surgical Endoscopy and other Interventional Techniques had the highest number of total citations and, *g* index and third greatest *m* index ranking. This had the greatest impact using this variable, followed by Surgical Endoscopy and other Interventional Techniques. The greatest number of total citations was in Surgical Endoscopy and Other Interventional Techniques, again followed by the Journal of Urology. 28% (*n* = 7) of the sources with the highest number of total citations were in the field of urology.

### Author-specific analysis

Table 4 shows author-specific results for the top 20 most productive authors. All the top 20 authors had a *h* index of at least 5 with a median (IQR) of 9.5 (398), and the number of total citations ranged from 71 to 1342, with a median (IQR) of 341.5(5.75). The median (IQR) *g* index was 15 (3.5) and scores ranged from 7 to 26. Furthermore, the median (IQR) *m* index was 0.61(0.27) with a range from 0.375 to 1.14.

**Table 4:** Author-specific results

Author	h Index	g Index	m Index	Total citations (TC)
Yang GZ	15	24	0.833	594
Stoyanov D	15	26	0.833	748
Kiaii B	10	14	0.588	233
Li J	5	9	0.385	96
Patel RV	9	18	0.600	328
Ahmad S	13	18	0.929	746
Holloway RW	15	18	1.071	810
Dasgupta P	7	16	0.412	275
Hubert J	8	17	0.421	355
Pigazzi A	11	17	0.688	1342
Wang Y	8	14	1.143	210
Kandil E	4	8	0.364	76
Patel VR	10	16	0.625	389
Darzi A	11	15	0.550	453
Fader AN	10	15	0.769	592
Gundeti MS	9	14	0.692	203
Kaouk JH	11	15	0.550	447
Kim S	5	13	0.556	169
Poignet P	6	10	0.375	119
Toloza EM	5	7	0.625	71

**Table 5:** Table showing statistics regarding the country of origin of the research

Country	Articles	Frequency	SCP	MCP	MCP/SCP Ratio
USA	1745	0.473028	1579	166	0.0951
China	275	0.074546	245	30	0.1091
Italy	209	0.056655	170	39	0.1866
Germany	180	0.048794	140	40	0.2222
United Kingdom	177	0.047980	133	44	0.2486
France	167	0.045270	138	29	0.1737
Canada	123	0.033342	95	28	0.2276
Japan	86	0.023313	77	9	0.1047
Australia	74	0.020060	61	13	0.1757
Korea	72	0.019517	59	13	0.1806
Turkey	67	0.018162	61	6	0.0896
India	56	0.015180	53	3	0.0536
Spain	56	0.015180	33	23	0.4107
Brazil	34	0.009217	23	11	0.3235
Belgium	30	0.008132	20	10	0.3333
Singapore	28	0.007590	20	8	0.2857
Switzerland	28	0.007590	20	8	0.2857
Israel	24	0.006506	16	8	0.3333
Denmark	23	0.006235	20	3	0.1304
Sweden	23	0.006235	17	6	0.2609
Romania	22	0.005964	20	2	0.0909
Greece	20	0.005422	16	4	0.2000
Netherlands	19	0.005150	13	6	0.3158
Austria	16	0.004337	13	3	0.1875
Iran	14	0.003795	13	1	0.0714
Saudi Arabia	14	0.003795	9	5	0.3571
Finland	12	0.003253	10	2	0.1667
Portugal	12	0.003253	7	5	0.4167
Ireland	11	0.002982	7	4	0.3636
Norway	11	0.002982	8	3	0.2727
Mexico	7	0.001898	6	1	0.1429
Egypt	6	0.001626	5	1	0.1667
Argentina	5	0.001355	4	1	0.2000
Chile	5	0.001355	4	1	0.2000
New Zealand	5	0.001355	5	0	0.0000
Thailand	5	0.001355	4	1	0.2000
United Arab Emirates	4	0.001084	3	1	0.2500
Colombia	3	0.000813	2	1	0.3333
Malaysia	3	0.000813	3	0	0.0000
Poland	3	0.000813	3	0	0.0000
Qatar	3	0.000813	1	2	0.6667
Czech Republic	2	0.000542	1	1	0.5000
Kuwait	2	0.000542	1	1	0.5000
Venezuela	2	0.000542	1	1	0.5000
Vietnam	2	0.000542	0	2	1.0000
Bulgaria	1	0.000271	1	0	0.0000
Indonesia	1	0.000271	0	1	1.0000
Luxembourg	1	0.000271	1	0	0.0000
South Africa	1	0.000271	1	0	0.0000

**Table 6:** Table showing number of occurrences of keywords

Words	Occurrences
Surgery	823
Outcomes	552
Experience	414
Cancer	304
Resection	271
Complications	231
Management	211
Learning-curve	192
Meta-analysis	138
Impact	133
System	118
Hysterectomy	117
Feasibility	104
Radical prostatectomy	104
Survival	103
Carcinoma	101
Lymphadenectomy	95
Children	91
Endometrial cancer	88
Trial	87
Laparotomy	86
Quality-of-life	86
Total mesorectal excision	82
Perioperative outcomes	75
Risk	74
Women	74
Initial experience	73
Accuracy	70
Laparoscopic surgery	69
Follow-up	67
Lymph-node dissection	67
Morbidity	67
Mortality	62
Tumours	62
Laparoscopy	60
Multicentre	59
Prostatectomy	59
Short-term outcomes	58
Replacement	57
Retropubic prostatectomy	57
Safety	57
Cost	55
Laparoscopic partial nephrectomy	54
Minimally invasive surgery	54
Repair	54
Performance	53
Rectal-cancer	50
Thoracic-surgery	49
Classification	47
Risk-factors	47

### Country-specific analysis

**Fig. 5:**
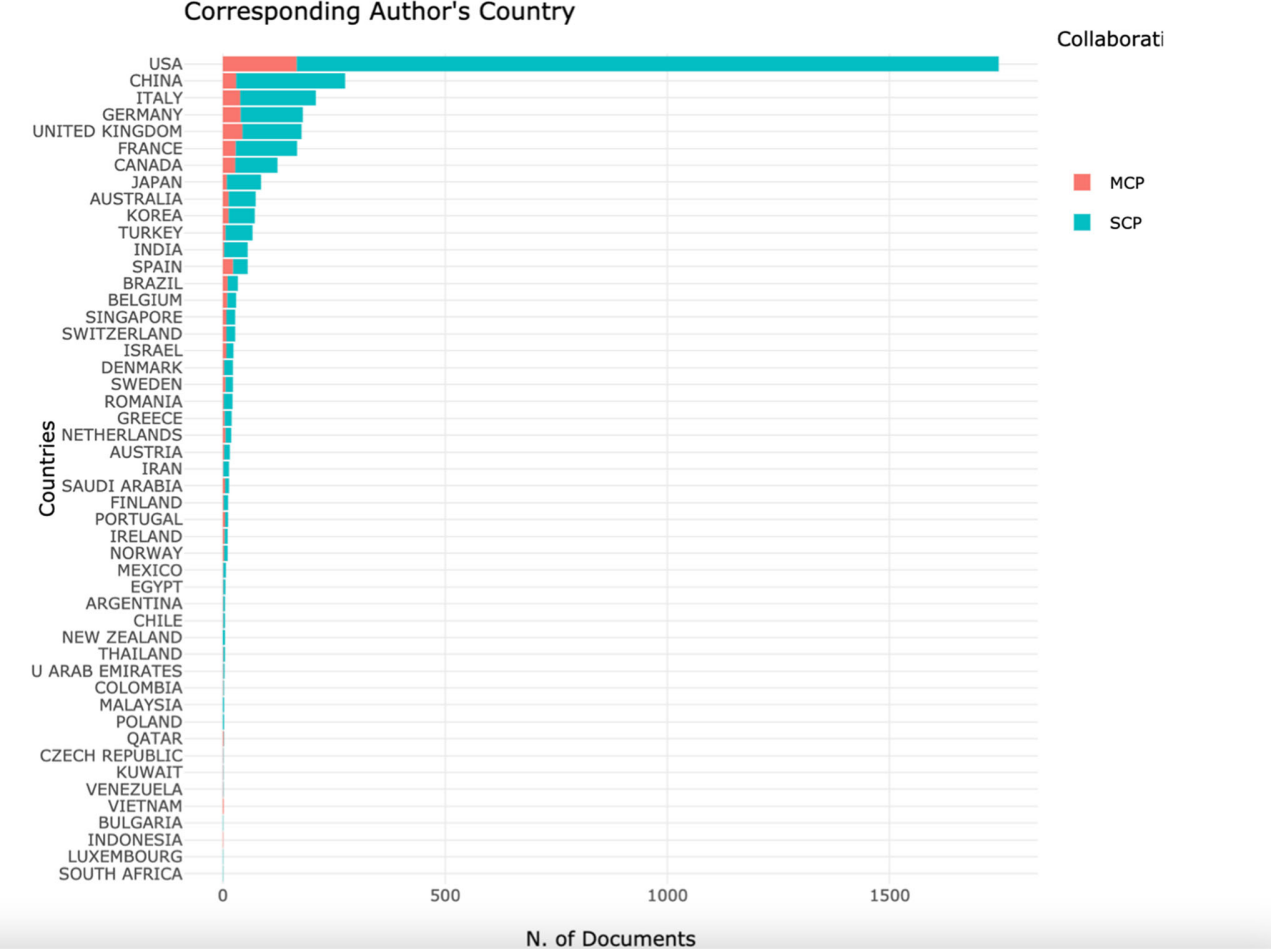
Graph showing the corresponding authors by countries

Figure 5 shows the spread of corresponding authors by country. As demonstrated, the USA had the highest number of articles (*n* = 1745), followed by China (*n* = 275) and Italy (*n* = 209). Table 4 then splits the data, including the SCP and MCP values.

**Fig. 6:**
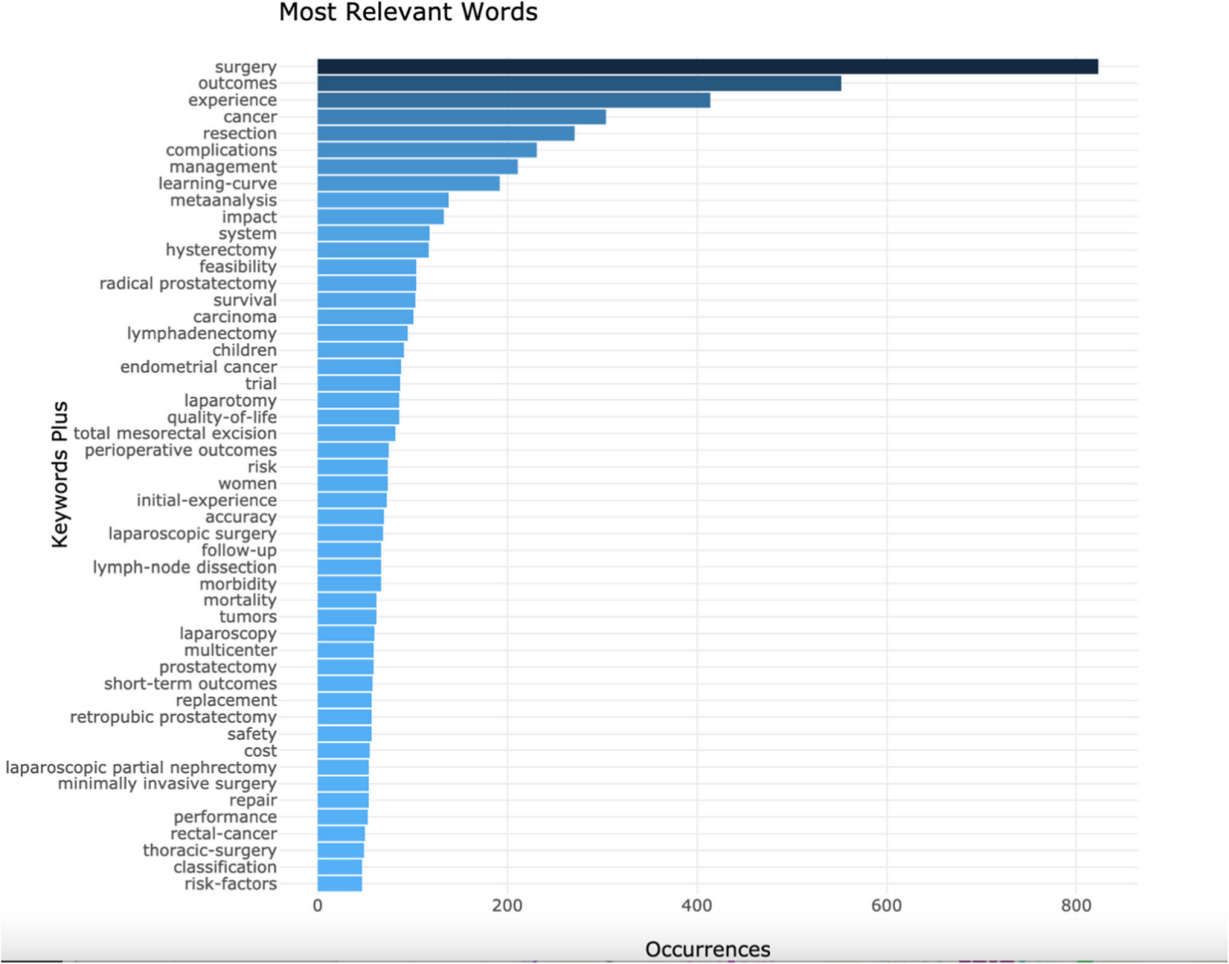
Diagram showing distribution of the top 50 keywords, based on their total number of occurrences

The median (IQR) number of articles per nation was 14 (51.25). When the documents were stratified by SCP and MCP, the median (IQR), respectively, was as follows: 13(29.25) and 3(8). The median (IQR) ratio of the two was 0.2 (0.208). Indonesia and Vietnam had the greatest international collaboration with an MCP/SCP of 1, followed by Qatar with 0.67, and Kuwait and Venezuela with 0.5 (Fig. 6).

### Keywords analysis

The most used word, as expected, was surgery with 823 occurrences. Outcomes (552) and experience (414) were the next two.

Twelve of the top 50 keywords were based on specific procedures: resection, hysterectomy, radical prostatectomy, lymphadenectomy, laparotomy, total mesorectal excision, laparoscopic surgery, lymph-node dissection, laparoscopy, prostatectomy, retropubic prostatectomy and laparoscopic partial nephrectomy. Other common themes were those relating to patient outcomes, such experience and quality-of-life.

**Fig. 7:**
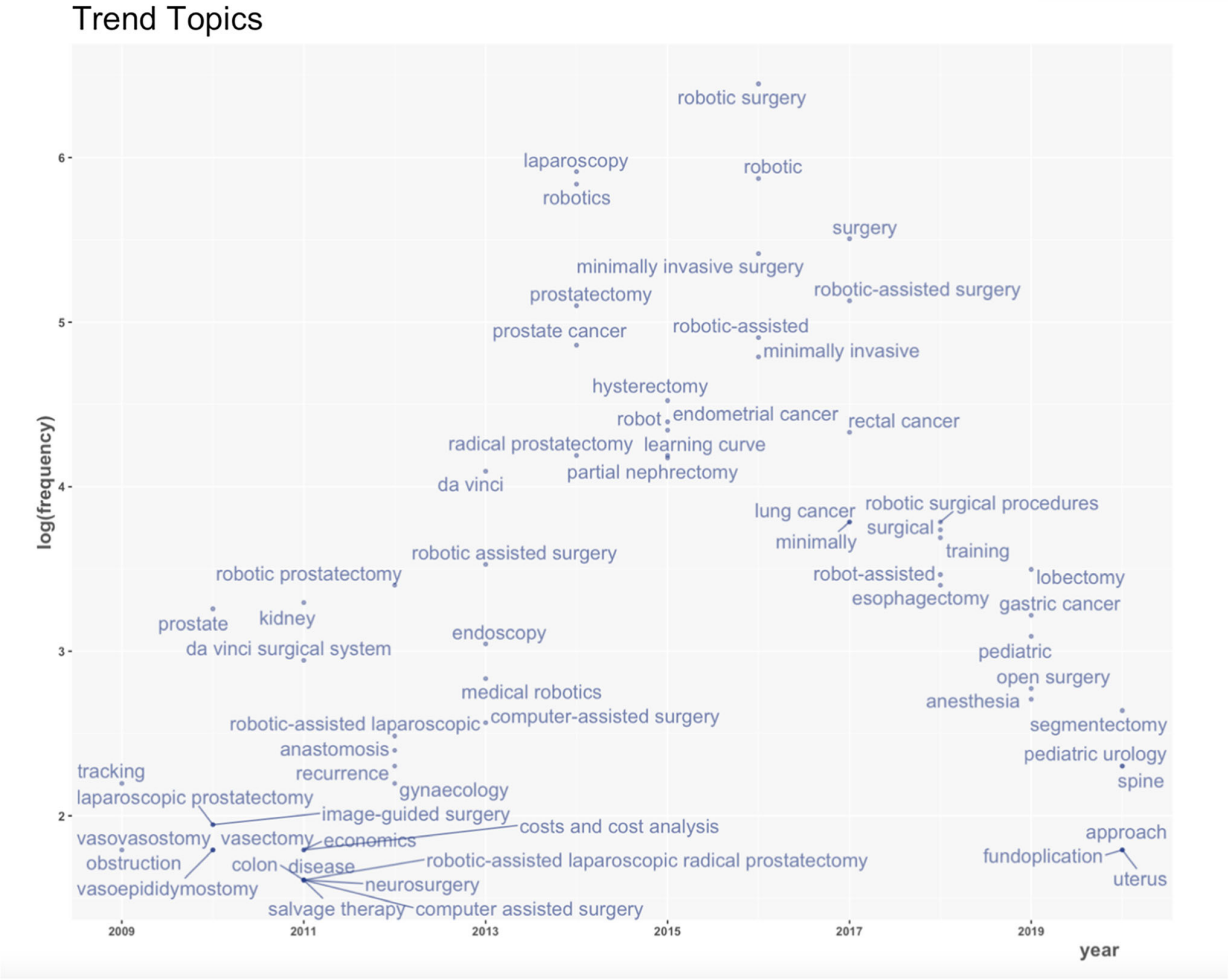
Graph showing the evolution of keywords from 2008 to 2021

Figure 7 shows how keyword frequency has changed from 2008 to 2021. Earlier publications focus on specific surgical techniques, such as conduit urinary diversion; however, overtime, keywords became more generalised with examples such as outcomes and accuracy in 2019–2020. Furthermore, as time moved towards the end of the decade, patient populations such as paediatrics came into consideration, along with different specialties, such as even Trauma and Orthopaedics, with the involvement of ‘spine’ as a keyword.

## Discussion

Research into robotic surgery is in its infancy but also changing and increasing. Over the past 20 years, over 3800 documents on the topic of robotic surgery have been compiled, gathering steam, especially from 2018 to 2020.

Despite this recent expansion, very few sources have fallen in the core of the literature, as shown by Bradford’s Law. Much of this research was in journals such as Urology, the Journal of Urology and the Journal of Minimally Invasive Gynecology, is related to urology or gynaecology, which has dominated the early stages of robotic research [12]. However, there has been a movement towards other specialties and subspecialties, such as general surgery and spinal surgery as shown by the keyword analysis.

Keyword analysis allows the reader to understand future frontiers of robotic surgery. The majority of research mentions surgical outcomes, complications and the importance of experience. If trends over time are considered, thoracic surgery and upper GI surgery are being increasingly mentioned in recent years, with keywords such as fundoplication and lobectomy cropping up during the final years of analysis.

When considering journal impact, the Journal of Robotic Surgery is the top-rated source through numerous metrics, such as relevance, Bradford Index, however, has only been active since 2007 and does not have an official impact factor, nor is it PubMed indexed.

Hirsch [13] hypothesised that after 20 years of research, a *H* index of 20 indicated a successful scientist, 40 indicated an outstanding scientist and 60 indicated extraordinary research output. As robotic research is still in its infancy, with few reaching even 20 years of research experience, thus the lack of any authors with a *H* index over 20 indicates the room for future research and growth in this field. The *m* index is a similar figure of the rate of productivity and shows a similar trend [14].

The most productive nations were the USA, China and Italy. This is expected, with the origins of robotic surgery beginning with Kwoh et al. [1] in California and market being dominated by the Da Vinci robotic system (Intuitive Surgical Inc, Sunnyvale, USA), originating in the United States, since 2000. Furthermore, The Business Research Company published a report in early 2020, indicating the North America was the largest region in the robotic surgical devices market in 2019, with Asia–Pacific growing at the greatest rate [15] (The Business Research Company, 2020). However, international collaboration levels remain low in these nations. Higher collaboration is present in nations with lower productivity. International collaboration has recently been realised with research such as the ROLARR trial [16], a product of collaboration between the United Kingdom, USA, Finland, Denmark, Italy and New Zealand.

### What does the future hold?

As mentioned earlier, collaboration may prove to be key. Using the vast potential of centres worldwide could lead to increased numbers of large-scale RCTs, allowing surgeons to explore different populations. Furthermore, there is a niche for further systematic reviews of the literature. As time passes, authors will be able to increase their productivity, with increased funding, leading to greater metrics, such as *H* index’s moving towards 20 + etc. Other areas of research, such as bariatric surgery, resectional surgery and spinal surgery also provide new frontiers.
